# Is it time to switch to doxycycline from azithromycin for treating genital chlamydial infections in women? Modelling the impact of autoinoculation from the gastrointestinal tract to the genital tract

**DOI:** 10.1186/s12879-015-0939-3

**Published:** 2015-04-30

**Authors:** Andrew P Craig, Fabian YS Kong, Laxmi Yeruva, Jane S Hocking, Roger G Rank, David P Wilson, Basil Donovan

**Affiliations:** The Kirby Institute, UNSW Australia, Sydney, NSW 2052 Australia; Centre for Epidemiology and Biostatistics, Melbourne School of Population and Global Health, University of Melbourne, Melbourne, Victoria 3004 Australia; University of Arkansas for Medical Sciences & Arkansas Children’s Hospital Research Institute, Little Rock, AR 72202 USA; Sydney Sexual Health Centre, Sydney Hospital, Sydney, NSW 2000 Australia

**Keywords:** *Chlamydia*, Azithromycin, Doxycycline, Re-infection

## Abstract

**Background:**

Single-dose azithromycin is recommended over multi-dose doxycycline as treatment for chlamydial infection. However, even with imperfect adherence, doxycycline is more effective in treating genital and rectal infection. Recently, it has been suggested that autoinoculation from the rectum to the genitals may be a source of persistent chlamydial infection in women. We estimated the impact autoinoculation may have on azithromycin and doxycycline effectiveness.

**Methods:**

We estimate treatment effectiveness using a simple mathematical model, incorporating data on azithromycin and doxycycline efficacy from recent meta-analyses, and data on prevalence of rectal infection in women with genital chlamydial infection.

**Results:**

When the possibility of autoinoculation is taken into account, we calculate that doxycycline effectiveness may be 97% compared to just 82% for azithromycin.

**Conclusions:**

Consideration should be given to re-evaluating azithromycin as the standard treatment for genital chlamydia in women.

## Background

Single-dose azithromycin has been recommended over a week-long doxycycline course as treatment for genital chlamydial infection, primarily because of concern about lack of adherence for the longer doxycycline course. However, the assumed superiority of azithromycin has been questioned [1]. In 2002, a meta-analysis concluded that azithromycin and doxycycline were equally efficacious in treating urogenital chlamydial infection (with efficacies of 97% and 98% respectively) [2], but a 2014 meta-analysis found a greater disparity, with efficacies of 94.3% for azithromycin and 97.1% for doxycycline [3]. For rectal infection, the difference may be greater: another systematic review estimated treatment efficacies of 82.9% for azithromycin and 99.6% for doxycycline [4], with different delivery mechanisms being suggested as a possible reason for the difference [5]. Additionally, background use of tetracyclines but not macrolides has been found to be associated with lower chlamydia prevalence [6].

It has recently been proposed that autoinoculation (the inoculation of a site with infective bodies from another site on the same individual) of chlamydia from the gastrointestinal (GI) tract to the genital tract is possible in women, and that the GI tract may be a niche for persistent infection [7-9]. Mice orally infected with chlamydia develop genital infections [10], similar to *Escherichia coli* urinary tract infections that occur in women as a result of faecal contamination, and rectal-vaginal autoinoculation is suspected to occur in infants [11]. It is at least theoretically possible, if not likely, that chlamydiae in the GI tract that have survived treatment with antibiotics may re-infect the genital tract in humans. Persistent infection or repeat infections in women are very common, with estimates of up to 29.9% among women reported [12], and are of concern because of the increased risk of pelvic inflammatory disease with repeat infection. If there is a substantial difference in the efficacy of doxycycline and azithromycin in resolving GI/rectal infection, and autoinoculation is a possibility, this may be further cause for reconsidering azithromycin as the preferred treatment for genital chlamydial infection.

We perform a simple calculation to estimate the probability that a woman with a genital chlamydial infection, treated with either azithromycin or doxycycline, remains chlamydia-free when the possibility of autoinoculation is considered. At present there are no estimates of the probability of autoinoculation from rectum to genital tract, so we consider the full range of probabilities from zero (autoinoculation never occurs) to one (autoinoculation always occurs). We consider two scenarios: the case of a woman known to be genitally infected but who has not been tested for rectal infection (as is usual practice), and the case of a woman known to have both genital and rectal infection.

## Methods

We use the random effects estimates of the efficacy of azithromycin and doxycycline from two recent systematic reviews: 94.3% for azithromycin and 97.1% for doxycycline against genital chlamydial infection [3], and 82.9% for azithromycin and 99.6% for doxycycline against rectal infection [4]. A subgroup analysis of just those studies that did not measure doxycycline compliance (and that therefore did not exclude any subjects based on low compliance levels) found a very similar random effects pooled estimate for difference in treatment efficacy to the estimate when all studies were included (1.4% and 1.5% respectively) [3], suggesting that these values are sufficiently close to real-world ‘use-effectiveness’ for the purposes of our study.

When women with genital chlamydial infection are tested for rectal infection, around 71-89% are positive [13-16]. Notably, there was no association with anal intercourse in those studies that reported it [14-16]. We assume 77% as the mean of the studies’ estimates. For the purposes of this study, we assume that the probability of genital infection cure, the probability of gastrointestinal tract infection cure, and the probability of autoinoculation are all independent.

If no rectal swab is taken, the probability that a woman remains free of genital infection after treatment can be estimated based on the probabilities of resolving genital and rectal infections and the probability of autoinoculation from the rectum to genitals; mathematically, the probability can be denoted by the expression:

$$ {P}_{genital}\left[1-\left(1-{P}_{rectal}\right){P}_{autoinoculation}{P}_{positive}\right], $$

where *P*_*genital*_ is the probability that a genital infection is resolved by treatment, *P*_*rectal*_ is the probability that a rectal infection is resolved by treatment, *P*_*autoinoculation*_ is the probability of autoinoculation occurring in a woman with a rectal infection, and *P*_*positive*_ is the probability of a woman with a genital infection also having a rectal infection. If the woman is known to have a rectal infection, the probability of remaining free of genital infection can be expressed mathematically as:

$$ {P}_{genital}\left[1-\left(1-{P}_{rectal}\right){P}_{autoinoculation}\right]. $$

## Results and discussion

**Figure 1:**
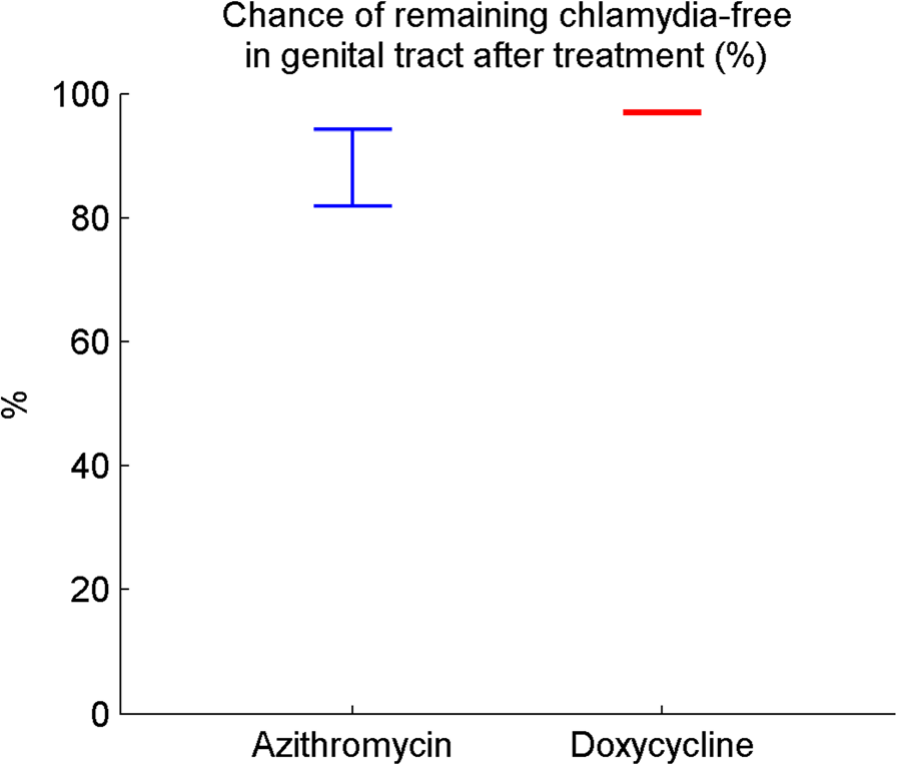
Chance of remaining chlamydia-free in genital tract after treatment. It is assumed that no rectal swab has been taken. The ranges reflect the range of probabilities for autoinoculation.

In Figure 1, we show the chance of the woman remaining free of genital chlamydial infection after treatment with either azithromycin or doxycycline, assuming that a rectal swab was not taken. The ranges reflect the autoinoculation probability varying from zero to one. When the probability of autoinoculation is one, the chance of the patient remaining free of genital infection after treatment with doxycycline is 96.8%, and 81.9% for azithromycin. That is, a 3.2% and 18.1% chance, respectively, of not clearing the infection. This corresponds to a 5.7-fold greater chance of not clearing an infection with azithromycin compared with doxycycline.

If the woman was known to have a rectal infection before treatment (i.e., if a positive rectal swab had been taken) then the ranges become wider: the chance of remaining free of genital infection after treatment with azithromycin is 78.2-94.3%, and after treatment with doxycycline 96.7-97.1%. This corresponds to a 2.0-6.6-fold greater chance of not clearing a chlamydial infection with azithromycin compared with doxycycline.

Using estimates from the literature of the efficacy of azithromycin and doxycycline in treating genital and rectal chlamydial infections, along with some simple assumptions, we have obtained estimates of the percentage chance of remaining free of chlamydia after treatment for genital chlamydia when the possibility of autoinoculation from rectum to genitals is taken into account. If autoinoculation does not occur, the efficacies of azithromycin and doxycycline are as reported in the systematic review (94.3% for azithromycin and 97.1% for doxycycline) and there is approximately a 2-fold greater chance of not clearing the infection with azithromycin. If autoinoculation has a high probability of occurring, then the efficacy of azithromycin may be as low as 81.9% when a patient’s rectal infection status is unknown, and as low as 78.2% if the patient is known before treatment to have a rectal infection. This means that the chance of not clearing an infection could be 6 times greater with azithromycin compared with doxycycline. It has usually been assumed that new infections detected after treatment for genital infection are due to re-infection by a partner, but it may be that some are due to autoinoculation. The disparity in efficacies provides further support for the careful re-evaluation of azithromycin as the preferred treatment for chlamydia.

Measuring the probability that autoinoculation from rectum to genitals occurs would be difficult. Assuming that there is some daily chance that autoinoculation occurs, whether autoinoculation has taken place would be a function of time since treatment. However, this is also the case for re-infection from a partner, and it would be difficult to separate the effects of these two mechanisms of re-infection. We recommend post-treatment tests for re-infection at both genital and rectal sites, as this will of course capture re-infection regardless of its source.

The ‘use-effectiveness’ of doxycycline does seem to be high, with a study that monitored adherence using microchipped medication bottles finding that chlamydial infection resolved in 76 of 81 (93.8%) of patients [17]. However, adherence did have an impact, with all 4 of the patients who failed therapy and were evaluable (i.e., returned their medication bottles) having at least two 24-hour intervals during which they did not take medication. Additionally, none of the evaluable 58 patients who took at least 10 doses failed therapy, while 4 of the evaluable 20 patients who took less than 10 doses failed therapy. While it seems that good use-effectiveness can be had from doxycycline, excessively low adherence is clearly to be avoided. Providers proscribing doxycycline for treatment of urogenital chlamydia should continue to encourage patients to take their full courses of medication.

## Conclusions

We have generated estimates of the percentage chance of a woman treated for genital chlamydia remaining free of genital infection after treatment, and found that this is much lower for azithromycin when autoinoculation from the rectum to the genitals is likely. A return to doxycycline as the standard treatment for chlamydial infection should be considered, and treatment trials of both genital and rectal infections should be encouraged.

## Abbreviation

GI: Gastrointestinal
